# Meta-analysis on the treatment of diabetic foot ulcers with autologous stem cells

**DOI:** 10.1186/s13287-017-0683-2

**Published:** 2017-10-16

**Authors:** Jianming Guo, Alan Dardik, Kacey Fang, Ruixue Huang, Yongquan Gu

**Affiliations:** 1 Department of Vascular Surgery, Xuanwu Hospital, Capital Medical University, Beijing, China; 2 Institute of Vascular Surgery, Capital Medical University, Beijing, China; 3 Section of Vascular Surgery, Vascular Biology and Therapeutics, Yale University, New Haven, CT USA; 4 Yale University, New Haven, CT USA; 5 Occupational and Environmental Health, Xiangya School of Public Health, Central South University, Hunan, China

**Keywords:** Diabetic foot, Meta-analysis, Randomized controlled trial, Stem cell, Ulcer

## Abstract

Over the last decade, many studies have indicated a therapeutic potential for treating diabetic lower extremity ulcers with autologous stem cells. The aim of the current study was to conduct a systematic review and meta-analysis of the treatment of diabetic foot ulcers (DFUs) with autologous stem cells. The search strategy included the Pubmed, EMBASE, Web of Science, and Cochrane’s Library databases. The endpoint measured was the healing of DFUs.

Six eligible randomized controlled trial (RCT) studies were screened from related published studies and reviewed for meta-analysis. The overall meta-analysis showed that stem cell administration was significantly favorable for healing diabetic ulcers (mean difference (MD) 0.52, 95% confidence interval (CI) 0.38–0.65; *p* < 0.00001). Subgroup analyses indicated that stem cells seemed to exert similar beneficial effects on patients with ulcer size ≥ 5 cm^2^ (MD 0.76, 95% CI 0.55–0.97; *p* < 0.00001) and < 5 cm^2^ (MD 0.43, 95% CI 0.31–0.54; *p* < 0.00001). Furthermore, stem cells had similar effects on patients aged ≥ 70 years (MD 0.61, 95% CI 0.14–1.08; *p* = 0.01) and < 70 years (MD 0.47, 95% CI 0.35–0.58; *p* < 0.00001). This systematic review and meta-analysis suggests a promising role for stem cells in DFU treatment. This review will pave the way to further study on the long-term effects of stem cell-based therapy and large-scale RCTs.

## Background

A diabetic foot is a foot with any pathology that results directly from diabetes mellitus (DM) or any of its chronic complications [1]. A diabetic foot is caused by neuropathy and/or peripheral arterial disease, especially with below-the-knee medium-to-small artery occlusions [2]. Diabetic foot ulcers (DFUs) occur in 7.2–15% of the diabetic population [3–5]. Between 5–24% of DFUs in patients will eventually lead to limb amputation within a period of 6–18 months after the first evaluation, and about 50% of amputees die within 5 years [1, 6, 7]. Considering the environment of multidisciplinary management, novel treatment strategies are needed to mitigate and overcome these disastrous diabetic complications.

The current standard DFU treatment protocol includes wound debridement, infection management, revascularization procedures when indicated, and ulcer off-loading [8]. However, given the wound healing pathological characteristics of multifactorial synergistic effects, most approaches have focused on one factor, such as inflammation or growth, which limits the therapeutic efficacy [9]. In addition, due to poor outflow, some DFU patients have no option for percutaneous or surgical revascularization. For these limitations, stem cell therapy has shown promise. Clinical and basic science studies show that cell therapies can provide a comprehensive solution by addressing multiple factors during diabetic wound healing, particularly for limb ischemic patients with no other options [10].

Some small-scale clinical trials have investigated the efficacy of applying stem cells to accelerate wound healing [11, 12]. However, the interpretation of the results may be biased by the limited statistical power and scale of the studies. On the other hand, although advanced age and ulcer size were found to have a negative effect on the healing of all diabetic foot ulcers in recent years [13–15], the effect of the age factor and ulcer size on efficacy of stem cells for treatment of diabetic ulcers is not clear. Therefore, we conducted a systematic review and meta-analysis of the published randomized controlled trials (RCTs) to evaluate the role of autologous stem cell administration in the treatment of DFUs.

## Methods

### Literature search

Two reviewers (JG and RH) independently searched the Pubmed, EMBASE, Web of Science, and Cochrane Library (Cochrane Center Register of Controlled Trials) databases, using the terms “stem cell(s)”, “progenitor cells”, “lipoaspirate cells” or “mononuclear cells” paired with “diabetic”, and “diabetes” paired with “wound”, “ulcer”, “foot” or “isch(a)emia”. The search was limited to clinical trial studies published in English. The final search was performed on 7 January 2017. Related original and review articles were identified manually, and references from these publications were also reviewed.

This systematic review and meta-analysis followed the Preferred Reporting Items for Systematic Reviews and Meta-analyses (PRISMA) criteria [16].

### Inclusion and exclusion criteria

Studies were included if: (1) they were reported as an RCT study; (2) they recruited patients with DFUs who were assigned to either a stem cell group that accepted autologous stem cell (derived from bone marrow, peripheral blood, umbilical cord blood, or adipose tissue) transplantation treatment, or a control group that accepted only placebo or other medical treatment; and (3) they reported outcomes regarding the healing of ulcers and where the relevant data could be estimated. Furthermore, cell therapy-related adverse events were also extracted. When the same groups of patients were reported in multiple papers, only the most recent and complete paper was selected to avoid overlap.

Studies were excluded if: (1) there was no control group in the study; (2) studies lacked measurement data; (3) the same test was repeated in published literature or subgroup analyses; and (4) they were non-English articles.

### Data extraction

According to the inclusion and exclusion criteria, all five authors (JG, RH, KF, AD, and YG) were involved in the literature search and data extraction. Quality was assessed according to the predefined inclusion criteria. Data regarding the first author, country of the study, publication date, characteristics of the included patients, number of participants, details of the stem cell therapy, follow-up duration, evaluation parameter such as healing rate, and incidences of adverse events were extracted.

### Statistical analysis

The analysis was performed with RevMan 5.3 software (the Cochrane Collaboration, 2014, Nordic Cochrane Center, Copenhagen, Denmark). Data were summarized as frequencies and continuous variables as standardized mean difference (SMD). The 95% confidence intervals (CIs) were analyzed as summary statistics. *I* ^2^ was used to evaluate interstudy heterogeneity. Both the fixed effects model and the random effects model were considered in the analysis depending on the *I* ^2^ result. If the study was not statistically homogeneous, we used a fixed effects model analysis; if there was heterogeneity between studies, we used a random effects model analysis. Funnel plots were used to test for publication bias. A two-tailed *p* value < 0.05 was considered statistically significant. From a clinical viewpoint, subgroup analyses were performed with the following factors: age (≥ 70 years or < 70 years) and ulcer size (≥ 5 cm^2^ or < 5 cm^2^).

## Results

### Search results and study characteristics

**Fig. 1:**
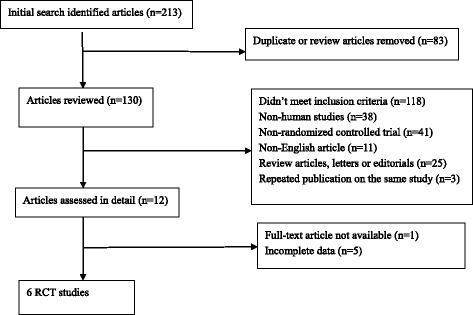
An adapted PRISMA flow diagram of the study selection process. RCT randomized controlled trial

An adapted PRISMA flow diagram [17] shows the literature screening process used in this report (Fig. 1). After careful review of the publications, six RCT studies were eligible and retrieved, including studies of bone marrow-derived mesenchymal stem cells (BMMSCs; *n* = 1), bone marrow-derived mononuclear cells (BMMNCs; *n* = 2), peripheral blood-derived mononuclear cells (PBMNCs; *n* = 1), bone marrow-enriched tissue repair cells (BMTRCs; enriched in CD90^+^ cells; *n* = 1), and human-processed lipoaspirate (PLA) cells (*n* = 1) [18–21]. Among the six studies, three were from China, two were from Germany, and one was from Korea. The characteristics of the studies are shown in Table 1.

**Table 1:** Characteristics of the included studies

Study	Mean age (years)	Participant cases (treatment group)	Stem cell type	Baseline ulcer size (cm^2^)	Cell number	Delivery method	Placebo	Follow-up duration (weeks)	Adverse events
Lu et al., 2011, China [18]	65	11	BMMNCs	4.3	NA	i.m.	N.S.	24	No
Lu et al., 2011, China [18]	63	11	BMMSCs	4.2	NA	i.m.	N.S.	24	No
Huang et al., 2005, China [19]	71	18	PBMNCs	2.7	3 × 10^9^/leg	i.m.	PGE1	12	No
Kirana et al., 2012, Germany [20]	69	12	BMMNCs	9.6	3 × 10^8^/leg	i.m.	No	45	No
Kirana et al., 2012, Germany [20]	71	12	BMTRCs	7.7	8 × 10^7^/leg	i.m.	No	45	No
Han et al., 2010, Korea [21]	67	26	PLA cells	4.3	> 4 × 10^6^/ulcer	Ad.us.ext.	No	8	No

*Ad.us.ext.* ad usum externum (for external use), *BMMNCs* bone marrow-derived mononuclear cells, *BMMSCs* bone marrow-derived mesenchymal stem cells, *BMTRCs* bone marrow-enriched tissue repair cells, *i.m.* intramuscularly, *NA* not available, *N.S* normal saline, *PBMNCs* peripheral blood-derived mononuclear cells, *PGE1* Prostaglandin E1, *PLA* human processed lipoaspirate

### Quality assessment

**Fig. 2:**
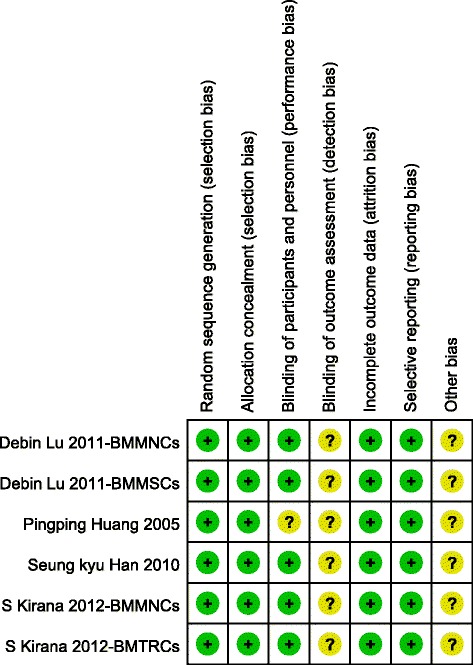
Risk of bias for each included RCT. *BMMNCs* bone marrow-derived mononuclear cells, *BMMSCs* bone marrow-derived mesenchymal stem cells, *BMTRCs* bone marrow-enriched tissue repair cells

The risk of biases of the included studies is shown in Fig. 2a and b. These data show that the highest risk of bias was in relation to performance and detection. Six RCT studies were included and the risk of bias for each RCT included was low.

### Effects of autologous stem cell therapy on the healing of lower extremity ulcers

**Fig. 3:**
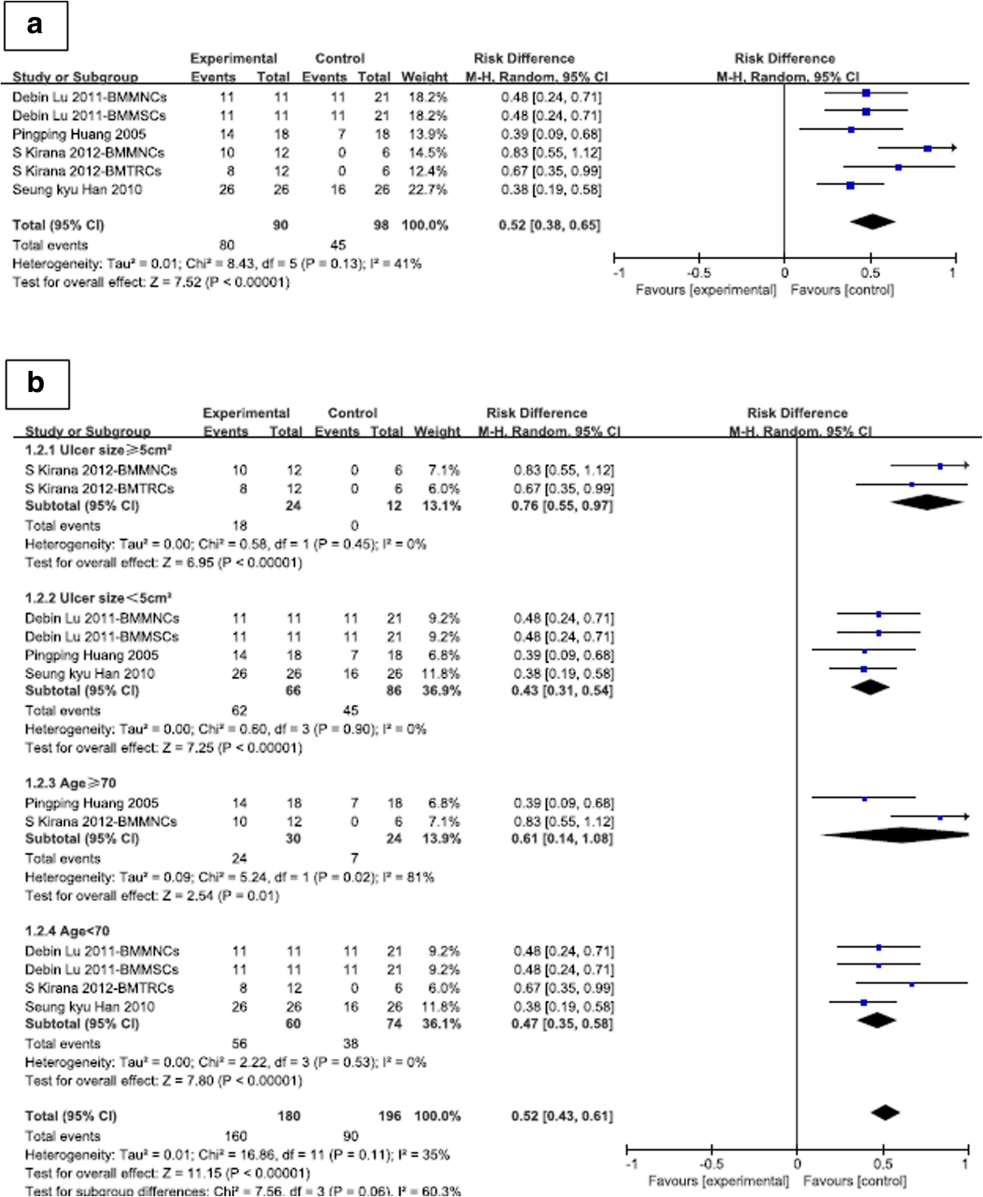
**a** Risk difference with stem cell therapy compared to control and interventions. **b** Risk difference with stem cell therapy compared to control and interventions by ulcer size and age group. *BMMNCs* bone marrow-derived mononuclear cells, *BMMSCs* bone marrow-derived mesenchymal stem cells, *BMTRCs* bone marrow-enriched tissue repair cells, *CI* confidence interval, *M-H* Mantel-Haenszel

As shown in Fig. 3a, the meta-analysis comparing the stem cell group to the control group showed a mean difference (MD) of 0.52 (95% CI 0.38–0.65; *p* < 0.00001), suggesting that stem cell-based therapy was associated with improved healing rate.

Figure 3b shows the results of the subgroup analysis based on ulcer size and age. The ulcer size was ≥ 5 cm^2^ in two of the studies (MD 0.76, 95% CI 0.55–0.97; *p* < 0.00001) and < 5 cm^2^ in four of the studies (MD 0.43, 95% CI 0.31–0.54; *p* < 0.00001). Two studies included patients ≥ 70 years (MD 0.61, 95% CI 0.14–1.08; *p* = 0.01) and four studies included patients < 70 years (MD 0.47, 95% CI 0.35–0.58; *p* < 0.00001).

The stability of the results was tested by sensitivity analysis which was conducted by showing that removing any one study would not affect the overall results of the effects of stem cell-based therapy. This suggests that any single study did not affect the overall results of the meta-analysis.

### Publication bias

**Fig. 4:**
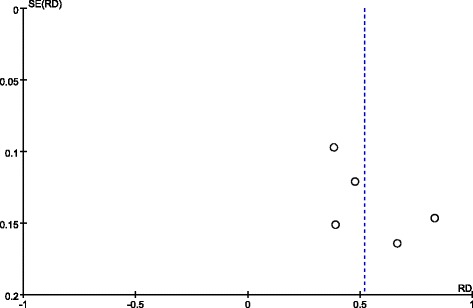
Funnel plot

Publication bias was qualitatively examined using a funnel plot. As shown in Fig. 4, the distribution of the funnel plot was nearly symmetrical, suggesting no obvious evidence of publication bias.

## Discussion

According to a report from the World Health Organization, an estimated 422 million adults were living with DM in 2016 [22]. In the United States, 9.3% of the population has diabetes [23]. In China, 11.6% of adults are diabetic, making China’s prevalence rate of DM the highest in the world [24]. The prevalence of diabetes is increasing rapidly; the prevalence among adults was high at 14.3% in the United States [25]. DFU is a major complication of DM, occurring in 15% of those with diabetes and preceding 84% of all diabetes-related lower leg amputations [4]. In the United States, DFUs represent a substantial cost burden among Medicare beneficiaries with diabetes, suggesting a 1-year cost of US$9–13 billion, in addition to the costs associated with the disease itself [26].

We present here the results of a systematic review and meta-analysis of RCTs complete with figures and a table (all original work from our team) where autologous stem cell treatment was administered to patients with DFUs. Our analysis suggests that stem cell treatment is safe and significantly helps diabetic ulcer healing, without any increased risk of treatment-related adverse events. To our knowledge, our study is the first meta-analysis evaluating the use of autologous stem cells as an effective treatment strategy for DFUs. The stem cell treatment was not associated with any increased risk of adverse events.

The prevalence of leg ulcers in the general population is 0.12%, but this rises to 1.2% in the population over 70 years of age. Two-thirds of pressure ulcers occur in patients who are over 70 years of age [27, 28]. Older adults with diabetes have significantly higher rates of major lower extremity amputation [29]. Considering the increasingly worsening general conditions and higher risks of traditional therapy, some older diabetic patients have a more immediate need for receiving stem cell treatment. However, advancing age negatively impacts stem cell function, and such age-related alterations may be detrimental for successful stem cell therapy [30, 31]. Duscher et al. [32] found that age-related changes in the mesenchymal stem cell population dynamics result in an impaired therapeutic potential of the aged progenitor cell. For older DFU patients, the efficacy of autologous stem cell transplantation is a clinical topic deserving attention. Our subgroup analysis suggests that stem cell treatment can effectively improve ulcer healing in diabetic patients < 70 years of age, as well as those ≥ 70 years of age. There are several possible reasons for this. First, biological functions of stem cells derived from an aged donor may be inferior to those derived from a younger donor, but both can enhance wound healing, especially in the hostile diabetic wound environment where many complicated factors may offset the difference in cells derived from aged and young donors. Second, only two research studies were included in our RCT subgroup of patients ≥ 70 years of age, where the mean ages were 70.9 years and 71.1 years. Given that both studies had a mean age close to the age endpoint of 70 years, this may have interfered with the accuracy of the RCT study results. Jiang et al. evaluated the effects of autologous stem cells on lower extremity ulcers and found autologous stem cell-based therapy was associated with better healing of lower extremity ulcers [33]; however, this study focused on patients with lower extremity ulcers that included diabetic patients and the non-diabetic population. Considering diabetes foot ulcers have specificity and distinctive risk factors, DFU patients should be seen as an independent observational target. Sun et al. conducted a meta-analysis concluding that applying autologous stem cell transplantation for curing limb ischemia does not show any obvious improvement in the limb ischemia, but that it can dramatically reduce the rate of amputation [34]. This meta-analysis has two shortages: one is that this study only assessed limb ischemia and did not discuss DFU; another is that this is an early study, published in 2015, where all the enrolled articles were published before 2012. As new studies on stem cells have been published more recently, the meta-analysis should be updated to give physicians more up-to-date information and conclusions. To our knowledge, our study is the first meta-analysis on the associations of diabetic foot ulcer treatment with autologous stem cells.

Ulcer size may have a negative effect on the healing of DFUs [1, 15]. The results of Skardal et al. [35] suggest that stem cells could be an effective treatment for large-scale wounds. In our analysis of the ulcer size subgroup, stem cell therapy had similar beneficial effects on the healing of both large and small ulcers (≥ 5 cm^2^ or < 5 cm^2^). This suggests that stem cell therapy may reduce the size of larger ulcers as well. Large diabetic cutaneous lesions typically have a poor blood supply, more serious tissue necrosis, inflammation, and bacterial contamination. The significant advantages of the application of regenerative therapies based on stem cells would be apt for this type of unfavorable environment [36]. As well as wound size, the wound depth, infection, and ischemia are also critical factors influencing wound healing [37]. Due to the limitations in article content, these parameters were not analyzed in our study.

The current study has several limitations. First, stem cell sources, the number of delivered cells, and the routes of cell administration differed among the studies. Due to these significant heterogeneities, optimized procedure protocols were not determined. Second, only the funnel plot was used to qualitatively assess publication bias, with no further examination by other methods such as Egger’s regression. Third, the claim about the older subgroup is not fully justified since the selected studies were performed on patients aged 63, 65, 67, 69, and 71 years. There is no remarkable difference between these ages. Looking forwards, we need to include more studies conducted on patients with a wider age range. Fourth, the RCTs were small in scale and size, and evidence from larger samples and more rigorous RCTs are required.

## Conclusion

This systematic review supports the promising role of stem cells in accelerating the healing of DFUs. Further evidence from larger, well-powered trials with long-term follow-up are needed to confirm our results.

## Abbreviations

BMMNC: Bone marrow-derived mononuclear cell
BMMSC: Bone marrow-derived mesenchymal stem cell
BMTRC: Bone marrow-enriched tissue repair cell
CI: Confidence interval
DFU: Diabetic foot ulcer
DM: Diabetes mellitus
MD: Mean difference
PBMNC: Peripheral blood-derived mononuclear cell
PLA: Processed lipoaspirate
PRISMA: Preferred reporting items for systematic reviews and Meta-analyses
RCT: Randomized controlled trial
